# Fasting and fasting-mimicking conditions in the cancer immunotherapy era

**DOI:** 10.1007/s13105-024-01020-3

**Published:** 2024-04-08

**Authors:** Ruben Pio, Yaiza Senent, Beatriz Tavira, Daniel Ajona

**Affiliations:** 1 Laboratory of Translational Oncology, Program in Solid Tumors, Cima Universidad de Navarra, Cancer Center Clínica Universidad de Navarra (CCUN), Pamplona, Spain; 2 Department of Biochemistry and Genetics, School of Sciences, Universidad de Navarra, Pamplona, Spain; 3 Navarra’s Health Research Institute (IDISNA), Pamplona, Spain; 4 Centro de Investigación Biomédica en Red Cáncer (CIBERONC), Madrid, Spain; 5 Department of Pathology, Anatomy and Physiology, School of Medicine, University of Navarra, Pamplona, Spain

**Keywords:** Fasting, Fasting-mimicking conditions, Tumor immunity, Cancer immunotherapy, Cancer treatment toxic side effects

## Abstract

Fasting and fasting-mimicking conditions modulate tumor metabolism and remodel the tumor microenvironment (TME), which could be exploited for the treatment of tumors. A body of evidence demonstrates that fasting and fasting-mimicking conditions can kill cancer cells, or sensitize them to the antitumor activity of standard-of-care drugs while protecting normal cells against their toxic side effects. Pre- and clinical data also suggest that immune responses are involved in these therapeutic effects. Therefore, there is increasing interest in evaluating the impact of fasting-like conditions in the efficacy of antitumor therapies based on the restoration or activation of antitumor immune responses. Here, we review the recent progress in the intersection of fasting-like conditions and current cancer treatments, with an emphasis on cancer immunotherapy.

## Background

Tumor progression requires the reprogramming of cellular energy metabolism in order to support continuous cell growth and proliferation [20]. This replacement of the metabolic program that operates in normal tissues have profound effects on gene expression, cellular differentiation and the tumor microenvironment (TME) [39] and may render cancer cells susceptible to metabolic intervention. Accordingly, a growing body of evidence suggests that nutrient deprivation, by fasting or fasting-mimicking conditions, may improve the use of anticancer agents. On the one hand, nutrient deprivation sensitizes cancer cells to the antitumor activity of a variety of cancer treatments [7, 27, 31, 52]. On the other hand, nutrient deprivation protects normal cells against stress and promotes tissue regeneration, attenuating the adverse side effects associated with cancer treatments[28, 29, 42]. Therefore, a reduction in caloric intake represents a promising strategy to improve the therapeutic effects of antitumor therapies. In any case, it is essential to balance this strategy with the recognition that maintaining adequate nutrition is indispensable for cancer patients dealing with challenges related to malnutrition and sarcopenia.

Cancer immunotherapy harnesses the immune system to generate an efficient anti-tumor immune response, which has led to remarkable responses and improved clinical outcomes in various types of cancer [50]. However, most patients do not respond to the treatment or acquire resistance [25]. Moreover, a proportion of patients experience severe immune-related adverse events (irAEs). In these cases, a dose reduction or the complete cessation of the treatment is required, limiting the antitumor efficacy of the treatment [24]. Therefore, novel strategies are needed to overcome the resistance of tumors to immunotherapy and to attenuate irAEs. Recently, our group and others have reported in preclinical cancer models that fasting or fasting mimicking conditions potentiate the antitumor response of cancer immunotherapies, and may hamper the magnitude of the irAEs associated with these treatments. This review will cover recent findings on the impact of dietary intervention in cancer therapy, with a special focus on cancer immunotherapy.

## Fasting and fasting-mimicking conditions as a promising approach to improve anticancer therapies

Fasting, which can be either continuous (caloric restriction) or intermittent (through a variety of fasting cycles)[55], improves the health and life span of laboratory animals [44]. In the clinic, a number of ongoing clinical trials are testing the impact of fasting or fasting-mimicking conditions in cancer patients. Clinical evidence suggests that a fast of at least 48 hours is required for attaining clinical effects [4, 11, 14]. To make this approach more bearable, a number of strategies have been developed to emulate fasting. Medically designed dietary regimes very low in calories, known as fasting mimicking diets (FMDs), have been developed to mimic the effects of fasting. Interestingly, the biochemical effects of fasting-like conditions can be also mimicked by caloric restriction mimetics (CRMs). CRMs are pharmacological compounds or natural agents that reduce lysine acetylation of cellular proteins and promote autophagy flux [40]. This deacetylation process can be achieved by compounds that reduce acetyl-coenzyme A levels, inhibit acetyl transferases or enhance the activity of deacetylases that reverse the action of acetyl transferases[15, 40].

Nutrient deprivation elicits an evolutionarily conserved molecular program characterized by modifications in the systemic levels of hormones and growth factors, such as insulin, glucagon, growth hormone, insulin-like growth factor 1 (IGF-1), glucocorticoids or adrenaline, which makes normal cells, but not cancer cells, more resistant to stressors [37]. Besides, various forms of reduced caloric intake, such as caloric restriction, fasting, FMD or the administration of CRMs, have proven effective in modulating tumor metabolism, remodeling the TME and enhancing the antitumor immune responses [55]. In accordance, a large number of preclinical studies have shown that fasting-like conditions reduce tumor growth, overcome treatment resistance, and mitigate adverse effects when combined with standard-of-care drugs [37]. Cyclic fasting/FMD promoted an increase in proteasome activity that served as a starvation escape pathway in chronic lymphocytic leukemia (CLL). Combining cyclic fasting/FMD with the proteasome inhibitor bortezomib and rituximab, an anti-CD20 antibody, slowed down CLL progression [43]. In the case of chemotherapy, the capacity of fasting or fasting-mimicking conditions to sensitize tumors to treatment has been attributed to the downregulation of IGF-1 and heme oxygenase 1 (HO-1) [13, 28, 29] an antioxidant protein with anti-apoptotic and cytoprotective effects via its catabolites as well as clearing toxic intracellular heme. A body of evidence demonstrates that HO-1 promote tumor progression and have immuno-modulatory roles that affect the tumor-associated immune infiltrate including Treg cells [35]. The upregulation of farnesyl-diphosphate farnesyltransferase-1 [52] and the induction of autophagy [40] have been also described as underlying mechanisms by which fasting or fasting-mimicking conditions sensitize tumors to chemotherapy. Interestingly, fasting-like conditions protect normal cells against the toxic side effects of chemotherapy [28, 29, 42]. Fasting-like conditions also potentiate the antitumor effects of hormone therapy, kinase inhibitors and ferroptosis inducers in preclinical models of cancer[7, 27, 31, 52]. In cancer patients, Vernieri *et al*. found that an FMD was safe, modulated the systemic metabolism and reshaped anticancer immunity by reducing the frequency of peripheral blood immunosuppressive regulatory T (Treg) cells, which paralleled with enhanced Th1/cytotoxic responses and an enrichment of IFNγ and other immune mediators associated with good clinical outcomes. FMD also ameliorated systemic myeloid immunosuppression, and reduced the proportions of total circulating CD14^+^ monocytes, M-MDSCs and PMN-MDSCs. Interestingly, FMD reduced the expression of CCL2 receptor (CD192) and fractalkine receptor (CX3CR1), mainly in the HLA-DR^−^ subset, indicating a reduction of proinflammatory/immunosuppressive functions. Likewise, there was an increase of monocytes expressing HLA-DR as well as of intermediate monocytes [49] which have been associated with clinical benefit in patients with melanoma treated with anti-PD-1 immunotherapy [26] and good prognosis in patients with breast cancer [51], respectively. Moreover, an increase of CD16^+^ DCs, a myeloid antigen-presenting cell population with enhanced T-cell priming ability [18], was observed [49]. Similar effects were previously described for fasting or fasting-like conditions in tumor-bearing mice. Fasting-mediated autophagy downregulated CD73 expression by tumor cells. As a consequence, the production of adenosine was decreased, preventing the shift of macrophages to an M2-like immunosuppressive phenotype [48]. An FMD in combination with chemotherapy increased the levels of common lymphoid progenitor cells (CLPs) in the bone marrow and of tumor-infiltrating CD8 T cells [13]. In the bone marrow compartment, dietary restriction also increased erythropoiesis, adipogenesis and memory T cell homing to enhance protection against tumors [8]. When combined with chemotherapy, fasting-mimicking conditions stimulated the hematopoietic system, induced a contraction of the Treg cell compartment, and enhanced immunosurveillance and CD8 T cell-mediated antitumor immune cytotoxicity [13, 40]. Therefore, there is a growing interest in evaluating the effects of fasting-like conditions in antitumor therapies based on the restoration or activation of antitumor immune responses.

## Utilizing fasting or fasting-mimicking conditions to improve the efficacy of cancer immunotherapy

The introduction of immunotherapy has represented a paradigm shift in the treatment of cancer. Since the approval of ipilimumab in 2011 for the treatment of melanoma, we have witnessed a remarkable progress in the development of cancer immunotherapies. There are currently dozens of approved immunotherapies and thousands of ongoing clinical trials. Current immunotherapy strategies include monoclonal antibodies against immune-regulatory molecules, adoptive cell-based therapies, vaccines, cytokines and oncolytic viruses, alone or combined with each other or with other treatment modalities[3]. Among them, immune checkpoint inhibitors are the most widely used cancer immunotherapies. These inhibitors work by blocking immune checkpoint proteins from binding with their partner proteins, allowing the immune cells to attack cancer cells more effectively. The most notable checkpoint inhibitors target programmed cell death protein 1 (PD-1), its ligand programmed death-ligand 1 (PD-L1) or cytotoxic T-lymphocyte-associated protein 4 (CTLA-4). These drugs have demonstrated remarkable success in various cancers, including melanoma, lung cancer, hepatocellular carcinoma and renal cell carcinoma[45]. However, only a small portion of those patients who receive these immune modulators will respond to the treatment. The etiologies of primary and acquired resistance to immunotherapy are multifaceted, and include the interplay between cancer and its TME, tumor immunogenicity (dependent on the tumor mutation profile, the tumor mutation burden and the patient's MHC variants), tumor heterogeneity, and patient's underlying immune status. Another major hurdle in the clinical application of immunotherapy is that a high proportion of patients develop irAEs associated with the hyperactivation of the immune system. These events range from mild to life-threatening, and are usually treated by discontinuing the administration of the therapeutic agents and/or by the application of temporary immunosuppression [41]. Therefore, although cancer immunotherapy has yielded remarkable improvements in clinical outcomes, the development of novel immunotherapy combination regimens, to overcome the hurdles described above, remains a major challenge [21].

Preclinical data suggest that dietary intervention improves the antitumor efficacy of cancer immunotherapy. In three syngeneic models of lung cancer (LLC, 393P and Lacun3), our group demonstrated that short-term starvation enhances anticancer immunosurveillance to facilitate the therapeutic activity of PD-1/PD-L1 blockade [1]. The therapeutic effect relied on the activity of CD8 T cells and, as previously observed for caloric restriction and FMD [13, 40] a reduction in the proportion of intratumoral Treg cells was observed. Interestingly, the therapeutic effect was linked to a decrease in circulating IGF-1 levels induced by caloric restriction. Accordingly, inhibition of the IGF-1R signaling on lung cancer cells reduced the proportion of tumor-infiltrating Treg cells and sensitized tumors to anti-PD-1 treatment to a similar extent as short-term starvation [1]. Moreover, high levels of plasma IGF-1 or IGF-1R expression on primary tumors were associated with resistance to anti-PD-1/PD-L1 therapy in patients with advanced non-small cell lung cancer[1]. IGF-1R signaling blockade also enhanced the antitumor effects of anti-PD-1 in combination with oxaliplatin in a mouse model of colorectal cancer[53]. These findings suggest that the IGF-1/IGF-1R pathway on tumor cells sustains an immunosuppressive TME that is involved in the primary resistance to PD-1/PD-L1 blockade. Although in this particular case the antitumor activity of short-term starvation was exerted by its direct effect on tumor cells, a body of evidence demonstrate the importance of metabolic alterations in the biology of the immune cells. The reduction of the circulating levels of growth factors, anabolic hormones, inflammatory cytokines and oxidative stress markers exerted by fasting and/or fasting-mimicking conditions [33] can affect the fate of immune cells. It is also important to consider the activity of fasting and/or fasting-mimicking conditions in the metabolism and the autophagy flux of immune cells because both dramatically influence the outcome of immune responses [2, 23]. Moreover, fasting and/or fasting-mimicking conditions can modify the microbiota composition and metabolism [32]. In a mouse model of colon adenocarcinoma, caloric restriction impaired tumor growth through a mechanism that is dependent on the gut microbiota. Mechanistically, *Bifidobacterium bifidum* mediated the caloric restriction-mediated antitumor effect through the acetate production and the accumulation CD8+ T cells in the tumor microenvironment [36]. The composition of the microbiote dramatically affects the efficacy of immunotherapy in cancer patients [5]. For instance, an increased relative abundance of *Bifidobacterium* was associated with more potent responses to anti-PD-L1 [46]. Therefore, the effects of fasting and/or fasting-like conditions in the microbiote could affect the efficacy of cancer immunotherapy. Thus, it is tempting to speculate that the effects of fasting and/or fasting-mimicking conditions in the composition of microbiote may affect the therapeutic activity of cancer immunotherapy.

**Fig. 1:**
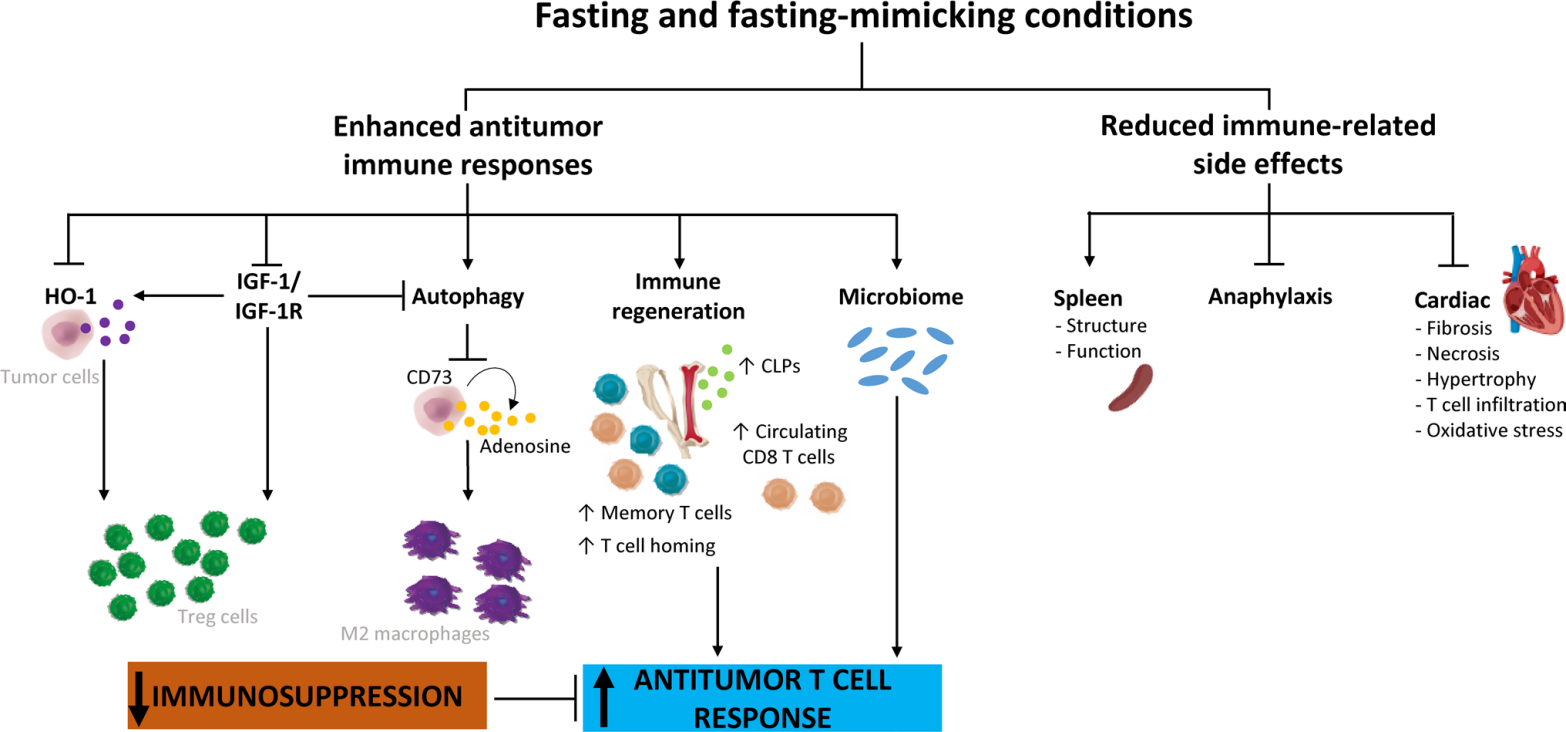
Summary of the mechanistic effects of fasting and fasting-mimicking conditions during cancer immunotherapy. Such interventions enhance the magnitude of the antitumor responses by the downregulation of HO-1 and IGF-1, the induction of autophagy, the reinvigoration of the cellular immunity, and the modification of the microbiote, whilst attenuating immune-related adverse events. Spleen and heart pictures were created with BioRender.com

Besides short-term starvation, other strategies have been proposed to reduce the caloric intake or mimic fasting conditions to improve the antitumor effects of cancer immunotherapy. In a fibrosarcoma syngeneic mouse model, the CRMs hydroxycitrate and spermidine potentiated the antitumor effect of a combined treatment based on immunogenic cell death-inducing agents and immune checkpoint inhibition [30]. Caloric restriction maintained OX40 agonist-mediated immunity against tumors and CD4 T cell priming during aging [16]. Other studies have supported the potential of utilizing dietary intervention to improve the antitumor efficacy of cancer immunotherapy. In mouse 4T1 breast tumors, an FMD reduced collagen accumulation within the tumor stroma, normalized the tumor vasculature network and promoted a metabolic shift from glycolysis to oxidative phosphorylation. Moreover, this FMD increased the efficacy of a combined anti-PD-L1/anti-OX40 treatment. The combined treatment was associated with a reactivation of effector T cells, an enhanced tumor-infiltration of natural killer cells and a reduction of tumor-infiltrating immunosuppressive leukocyte subsets, such as M2-polarized macrophages and Treg cells. The FMD also preserved the spleen structure and functionality and reduced the risk of anaphylaxis associated with immunotherapy [9]. FMD cycles, alone or in combination with a combined anti-PD-L1/anti-OX40 treatment, were also more effective than immune checkpoint inhibitors alone in delaying melanoma and lung growth in mice [10]. Moreover, the FMD cycles prevented the cardiac fibrosis, necrosis and hypertrophy caused by immune checkpoint inhibitors. This protective effect was associated with a reduction of T cell infiltration in myocardial tissues and of circulating and myocardial markers of oxidative stress and inflammation [10]. Figure 1 summarizes the mechanisms by which short-term starvation, fasting-mimicking conditions or dietary interventions benefits cancer immunotherapy.

Besides short-term starvation and FMD, a variety of other dietary regimens have been shown to increase the efficacy of immune checkpoint inhibitors, including ketogenic [17], protein restricted [38] and high fiber [47] diets. Supplementation or deprivation of specific nutrients has also been proposed as a strategy to potentiate the antitumor efficacy of immunotherapy. High-doses of ascorbic acid synergized with anti-PD-1 in a lymphoma mouse model [34]. Vitamin D supplementation increased the objective response rate and prolonged progression-free survival in patients with advanced melanoma undergoing anti-PD-1 therapy [19]. Similarly, supplementation with vitamin B5 increased the efficacy of PD-L1-targeted cancer immunotherapy [6]. Inhibition of retinoic acid activity in tumors enhanced the number of stimulatory monocyte-derived cells, enhanced T cell-dependent anti-tumor immunity, and synergized with immune checkpoint blockade[12]. Intermittent methionine deprivation sensitized tumor cells against CD8 T cell-mediated cytotoxicity and synergized with anti-PD-1 immunotherapy by the regulation of cation transport regulator homolog 1 (CHAC1) [54]. In contrast, methionine restriction impaired the tumor response to anti-PD-1 in a colon carcinoma mouse model [22], suggesting that the impact of methionine in cancer immunotherapy is context-dependent. Taken together, these findings suggest that the availability of nutrients plays a major role in tumor immunity. Combinatory treatments of cancer immunotherapy with optimized diet regimens own great potential to improve antitumor immune responses.

## Concluding remarks

Compelling evidence demonstrates that nutrition has a considerable influence on both the incidence and progression of cancer. Preclinical and clinical data suggest that fasting or fasting-mimicking conditions enhance the efficacy of current cancer therapies. These dietary interventions generate environments that disrupt oncogenic metabolic routes involved in the primary or acquired resistance to treatment, and protect non-malignant tissues from the side effects caused by antitumor drugs. Moreover, fasting or fasting-mimicking conditions regenerate antitumor immunity and prevent the immunosuppression caused by anticancer drugs. Therefore, in the current rush to advance cancer immunotherapy, the combination of fasting or fasting-mimicking conditions with cancer immunotherapy has been extensively used preclinically to enhance the efficacy of therapies based on the stimulation of the antitumor immunity. Moreover, some clinical data suggest the applicability of these findings to humans. However, there are still major challenges in the field, including the optimization of the fasting protocols, the delineation of the molecular mechanisms underlying the antitumor effects of fasting or fasting-mimicking conditions, the discovery of therapeutic targets associated with fasting or fasting-mimicking conditions, and the identification of those tumors that are most likely to be sensitive to these dietary interventions. In addition, the clinical application of fasting and fasting-mimicking conditions should be undertaken with caution in order to avoid undesired effects in cancer patients at nutritional risk. The biologically-relevant interplay between fasting or fasting-mimicking conditions and current cancer treatments encourages the design of future studies to maximize efficacy and achieve the lowest toxicity of current cancer treatments.

## Data Availability

Data generated or analyzed during this study are available from the corresponding author on reasonable request.
